# Data set of enzyme fingerprinting of dietary fibre components (arabinoxylan and β-glucan) in old and modern Italian durum wheat genotypes

**DOI:** 10.1016/j.dib.2017.12.029

**Published:** 2017-12-19

**Authors:** Michele A. De Santis, Ondrej Kosik, Diana Passmore, Zina Flagella, Peter R. Shewry, Alison Lovegrove

**Affiliations:** a Dipartimento di Scienze Agrarie, degli Alimenti e dell’Ambiente (SAFE), Università degli Studi di Foggia, Via Napoli 25, 71122 Foggia, Italy; b Rothamsted Research, Harpenden, Hertfordshire AL5 2JQ, United Kingdom

## Abstract

The data presented are related to the research article entitled “Comparison of the dietary fibre composition of old and modern durum wheat (*Triticum turgidum* spp. *durum*) genotypes” (De Santis et al., 2018) [1]. This article provides details of the structures of the major dietary fibre components, arabinoxylan and β-glucan, in semolina and wholemeal flour of old and modern Italian durum wheat genotypes grown in two seasons, determined by enzyme digestion followed by high-performance anion-exchange chromatography (enzyme fingerprinting).

**Specifications Table**

**Table:** 

Subject area	*Agriculture and Biological sciences*
More specific subject area	*Genetic differences and Food Quality*
Type of data	*Tables*
How data was acquired	*Laboratory analysis by High-Performance Anion-Exchange Chromatography (HP-AEC)*
Data format	*Raw, analyzed*
Experimental factors	*The structures of dietary fibre components were determined in wholemeal and semolina from old and recent Italian durum wheat genotypes grown in two field trials*
Experimental features	*Allowed the identification of relationships between fibre structure and the release dates of the genotypes*
Data source location	*Foggia (Italy, 41° 28′ N, 15° 32′E and 75 m a.s.l.), collected in June 2013 and June 2014*
Data accessibility	*Data are available in this article*

**Value of the data**

• Details of the structures of arabinoxylan and β-glucan determined by the proportions of arabinoxylan oligo-saccharides (AXOS) and glucooligosaccharides (GOS) released by digestion with xylanase 11 and lichenase, respectively
• Allows comparison of old and recent types of durum wheat: 8 modern cultivars, 3 old cultivars bred before 1949 and 4 old landraces
• Includes comparison of wholemeal and white flour (semolina) fractions

## 1 Data

The datasets provide details of the structure of dietary fibre in grains of old and modern Italian durum wheat (Triticum turgidum spp. durum) genotypes, grown in two different crop seasons [1]. Table 1 presents the grain quality traits of the genotypes grown in two seasons while Table 2, Table 3 give details of the structures of arabinoxylan and β-glucan determined by enzymatic fingerprinting of semolina and wholemeal flours. Correlations between grain quality parameters and dietary fibre content and composition are reported in Table 4.

**Table 1:** Grain quality traits of old and modern durum wheat genotypes grown in two crop seasons.

Genotype	Year of release	1000 kernel weight (g)		Test weight (kg hl^-1^)		Grain protein content (% dm)		Semolina protein content (% dm)		Ash content (% dm)	
		2013	2014	2013	2014	2013	2014	2013	2014	2013	2014

*old*											
Dauno III	1900	47.8	45.8	77.4	78.3	15.9	14.3	14.0	13.1	0.77	0.80
old Saragolla	1900	49.2	49.1	80.7	76.7	15.4	16.4	13.6	15.3	0.80	0.76
Russello	1910	48.6	54.9	78.7	79.1	16.5	13.6	14.5	12.3	0.85	0.88
Timilia R.B.	1910	35.8	34.5	80.6	79.2	16.4	13.9	14.8	12.8	0.70	0.88
Cappelli	1915	54.4	48.6	81.3	79.2	16.8	14.5	15.3	13.5	0.82	0.80
Garigliano	1927	56.0	64.1	78.9	76.1	15.4	14.9	13.8	14.3	0.83	0.80
Grifoni 235	1949	53.4	54.9	80.4	75.6	15.6	13.1	12.0	11.1	0.79	0.86
*modern*											
Adamello	1985	55.6	37.8	79.6	67.5	14.0	16.0	12.5	14.8	0.83	0.87
Simeto	1988	47.7	38.3	79.8	69.6	15.4	13.4	14.0	12.1	0.60	0.82
Preco	1995	57.2	31.7	81.6	66.7	13.0	15.9	11.6	14.6	0.87	0.96
Iride	1996	54.1	36.0	82.2	80.5	13.0	11.5	11.3	10.8	0.60	0.86
Svevo	1996	45.8	33.6	82.2	73.6	16.1	15.0	14.8	13.8	0.82	0.73
Claudio	1998	50.8	41.6	84.5	81.8	12.4	11.7	10.6	10.8	0.83	0.60
Saragolla	2004	38.0	43.8	83.0	79.9	12.7	12.0	11.2	11.2	0.74	0.85
PR22D89	2005	57.0	41.4	83.8	74.7	12.6	12.9	10.7	12.2	0.80	0.87
LSD⁎		0.64		0.09		0.04		0.07		0.005	

⁎ Least significant difference (LSD) at *P* ≤ 0.05.

**Table 2:** Structures of dietary fibre components in semolina from old and modern Italian durum wheat genotypes, grown in two crop seasons, determined as percentages of AXOS and GOS from enzyme fingerprinting.

Genotypes	x		x_2_		x_3_		x_5_		xa^3^xx		xa^3^a^3^xx		xa^3^xa^3^xx		xa^2+3^xx		xa^3^a^2+3^xx		xa^3^xa^2+3^xx		G3:G4		β-glucan peak area	
	(%)		(%)		(%)		(%)		(%)		(%)		(%)		(%)		(%)		(%)		(ratio)		(nC)	
	2013	2014	2013	2014	2013	2014	2013	2014	2013	2014	2013	2014	2013	2014	2013	2014	2013	2014	2013	2014	2013	2014	2013	2014

*Old*																								
Dauno III	17.5	17.5	18.0	16.0	8.2	8.7	12.6	11.0	13.9	15.0	6.1	6.1	2.4	2.6	14.8	15.9	4.0	4.5	2.5	2.6	2.05	2.57	14,776	12,227
old Saragolla	19.7	22.3	19.8	17.2	8.3	6.9	5.6	4.0	13.6	15.6	6.0	5.5	2.6	2.9	17.2	17.5	4.2	4.9	2.9	3.2	2.25	3.08	14,041	10,262
Russello	19.5	19.3	19.0	15.6	5.5	5.3	11.3	9.9	14.2	16.4	8.0	8.0	2.3	2.6	14.0	15.7	3.7	4.3	2.6	2.8	2.40	2.74	13,027	15,141
Timilia R.B.	19.7	18.7	14.2	12.7	6.8	7.1	9.5	7.5	15.9	17.1	9.1	9.1	2.8	3.1	15.7	17.0	3.8	4.6	2.6	3.0	2.39	2.97	13,011	18,224
Cappelli	18.9	19.9	13.2	16.1	7.6	5.3	13.0	10.1	15.5	14.8	6.7	6.4	2.4	2.4	16.1	17.4	3.9	4.8	2.7	3.0	2.04	2.26	14,414	10,824
Garigliano	20.5	17.8	15.9	15.3	4.4	6.7	10.9	10.1	13.5	15.4	6.7	6.5	2.0	2.5	18.4	18.1	4.6	4.7	3.1	2.9	2.40	2.62	8,272	14,117
Grifoni 235	19.0	18.7	11.5	13.3	4.2	4.4	14.1	10.7	11.9	13.2	6.0	6.0	2.5	2.7	22.1	21.5	5.8	5.9	3.3	3.4	1. 82	2.86	17,720	15,641
*modern*																								
Adamello	19.3	20.7	12.9	16.1	6.3	7.3	15.6	10.9	13.8	13.9	5.0	4.1	2.0	2.0	17.1	16.6	4.8	4.9	3.3	3.4	2.30	3.85	16,137	9,224
Simeto	21.4	19.1	15.4	12.2	6.3	5.9	6.7	13.1	15.5	14.7	5.5	5.2	2.2	2.5	18.3	18.9	5.1	5.1	3.5	3.3	2.12	2.79	12,152	10,389
Preco	17.9	20.4	15.6	16.8	10.2	7.8	13.3	9.3	11.6	13.6	6.7	5.1	2.3	2.0	15.6	16.8	4.0	5.0	2.8	3.3	2.22	3.16	14,297	14,460
Iride	18.4	18.9	15.5	15.2	5.6	7.2	14.1	14.3	12.8	14.4	6.9	5.7	2.5	2.1	16.6	14.6	4.3	4.4	3.4	3.3	1.98	2.72	16,059	21,937
Svevo	19.2	20.5	17.6	14.7	6.8	8.0	12.8	12.8	13.5	13.6	4.5	5.1	1.7	1.7	16.6	15.6	4.3	4.6	3.2	3.5	2.20	2.98	19,808	15,693
Claudio	17.8	17.0	13.8	12.2	6.1	6.4	14.6	18.0	14.7	14.4	7.1	6.3	2.4	2.4	16.1	15.7	4.5	4.5	3.1	3.0	2.17	2.73	23,185	22,402
Saragolla	16.1	18.2	12.9	12.9	5.0	7.4	14.2	14.6	14.8	15.0	7.7	6.6	2.5	2.7	17.8	14.9	5.1	4.5	3.7	3.2	2.02	2.64	25,275	21,052
PR22D89	19.0	17.4	11.2	14.3	6.2	8.5	13.4	13.9	16.1	14.7	5.7	5.2	2.2	2.1	18.6	15.8	4.5	4.8	3.0	3.2	2.17	2.71	21,540	20,181
⁎LSD	0.28		0.85		0.25		0.36		0.24		0.15		0.07		0.26		0.08		0.05		0.06		1,163	

⁎ Least Significant Difference (LSD) at *P* ≤ 0.05.

**Table 3:** Structures of dietary fibre components in wholemeal flours from old and modern Italian durum wheat genotypes, grown in two crop seasons, determined as percentages of AXOS and GOS from enzyme fingerprinting.

Genotype	x		x_2_		x_3_		x_5_		xa^3^xx		xa^3^a^3^xx		xa^3^xa^3^xx		xa^2+3^xx		xa^3^a^2+3^xx		xa^3^xa^2+3^xx		G3:G4		β-glucan peak are	
	(%)		(%)		(%)		(%)		(%)		(%)		(%)		(%)		(%)		(%)		(ratio)		(nC)	
	2013	2014	2013	2014	2013	2014	2013	2014	2013	2014	2013	2014	2013	2014	2013	2014	2013	2014	2013	2014	2013	2014	2013	2014

*old*																								
Dauno III	25.1	23.9	19.6	37.3	10.0	7.5	5.0	4.3	17.6	11.7	4.1	2.6	1.2	0.7	11.3	7.7	3.0	1.9	3.2	2.4	2.30	2.82	39,190	21,236
old Saragolla	25.1	22.7	21.6	36.9	9.5	8.1	3.6	2.0	16.3	13.0	3.2	2.1	1.2	1.0	12.4	9.1	3.0	2.2	4.0	2.7	2.30	3.36	38,278	18,285
Russello	25.6	27.3	21.2	30.9	9.3	6.5	5.0	4.6	15.9	12.3	4.1	3.8	1.0	0.9	11.3	9.1	3.0	2.1	3.6	2.4	2.44	2.72	40,881	23,568
Timilia R.B.	27.0	20.8	21.5	35.9	7.9	9.4	3.5	4.1	18.1	13.3	4.0	3.8	1.0	0.9	10.4	8.1	2.7	1.9	3.3	2.4	2.42	2.84	55,019	25,195
Cappelli	26.3	21.6	20.9	36.2	10.7	8.8	6.0	4.4	15.3	11.6	3.4	2.7	1.0	0.8	10.7	9.1	2.7	2.1	3.0	2.5	2.36	3.79	30,867	14,613
Garigliano	25.6	21.4	21.5	33.9	7.5	7.4	5.5	4.8	16.3	13.3	3.8	3.0	0.9	0.9	12.5	10.4	2.9	2.3	3.4	2.5	2.70	3.11	33,440	20,228
Grifoni 235	26.0	21.5	18.9	33.1	7.9	7.2	7.3	4.4	15.6	13.1	3.4	2.9	1.0	0.9	13.2	11.4	3.3	2.7	3.4	2.6	2.27	3.97	33,774	23,406
*modern*																								
Adamello	27.5	22.7	20.2	39.6	9.0	7.9	6.3	4.0	15.2	10.7	2.6	1.5	0.9	0.7	11.9	8.3	3.0	2.2	3.3	2.5	2.46	5.71	41,590	23,206
Simeto	22.7	28.6	36.7	23.6	10.2	7.2	2.1	4.4	13.4	14.3	2.1	2.5	1.1	0.9	9.7	10.1	2.3	2.6	2.6	2.9	2.56	4.84	21,980	32.461
Preco	24.3	21.9	18.2	37.9	8.4	7.6	5.7	3.0	15.5	11.3	3.9	2.5	1.2	1.7	12.3	9.2	3.2	2.5	7.3	2.5	2.43	3.98	63,886	22,179
Iride	28.5	20.4	24.7	39.1	7.3	9.0	4.5	5.8	15.5	10.5	3.2	1.9	0.9	0.7	9.9	7.4	2.6	2.1	3.3	2.8	2.52	2.80	47,092	33,305
Svevo	26.4	21.8	23.1	37.8	7.4	8.0	4.9	4.1	16.2	10.9	2.6	1.9	0.9	0.9	11.7	9.4	3.1	2.5	3.6	2.6	2.60	3.35	51,950	29,761
Claudio	23.9	21.4	21.7	35.8	10.8	7.3	7.0	5.7	16.2	11.9	3.3	2.6	0.9	1.0	10.0	9.0	2.9	2.5	3.3	2.7	2.52	2.97	60,886	30,866
Saragolla	28.2	20.9	22.9	36.7	8.3	7.7	4.2	5.3	14.9	11.3	2.9	3.1	1.0	1.0	11.1	9.0	3.0	2.3	3.4	3.0	2.55	2.79	52,471	33,626
PR22D89	25.6	20.2	20.3	34.9	8.8	9.2	4.8	4.2	16.8	12.5	3.3	2.8	1.0	0.9	12.5	10.2	3.2	2.5	3.5	2.5	2.46	3.57	57,894	24,381
⁎LSD	1.08		0.78		0.53		0.21		0.37		0.11		0.04		0.34		0.09		0.43		0.10		3,075	

⁎ Least significant difference (LSD) at P ≤ 0.05.

**Table 4:** Correlation matrix of the main kernel quality parameters with year of release and with the content and composition of arabinoxylan (AX) and β-glucan in semolina and wholemeal flours of old and modern durum wheat genotypes.

	YR	TKW	TW	Tot-AX	WE-AX	RV
*Grain parameters*						
TKW	−0.24	1.00	0.42	−0.06	−0.30	−0.37
TW	−0.02	0.42	1.00	−0.34	−0.76	−0.73
GPC	−0.50	−0.08	−0.27	−0.36	−0.33	–
SPC	−0.48	−0.18	−0.33	−0.04	−0.20	−0.48
Ash	−0.06	0.03	−0.21	0.36	−0.17	−0.19
						
*Semolina fibre components*						
Tot-AX	0.05	−0.06	−0.34	1.00	0.44	0.27
WE-AX	−0.09	−0.30	−0.76	0.44	1.00	0.78
AX solubility	−0.17	−0.29	−0.68	0.04	0.90	0.72
RV	0.14	−0.37	−0.73	0.27	0.78	1.00
%x	−0.20	−0.07	−0.36	0.08	0.19	0.23
%x_2_	−0.36	0.02	−0.14	0.05	0.09	0.11
%x_3_	0.03	−0.23	−0.16	−0.02	0.27	0.47
%x_5_	0.60	0.02	0.26	−0.05	−0.22	−0.17
%xa^3^xx	−0.29	−0.16	0.07	−0.18	0.04	−0.08
%xa^2+3^xx	−0.01	0.30	−0.08	0.13	−0.03	−0.11
%xa^3^a^3^xx	−0.41	0.05	0.45	−0.14	−0.19	−0.34
%xa^3^xa^3^xx	−0.51	0.08	0.15	−0.04	−0.06	−0.30
%xa^3^a^2+3^xx	0.24	−0.06	−0.34	0.18	0.17	0.06
%xa^3^xa^2+3^xx	0.64	−0.28	−0.21	0.08	−0.01	0.10
G3: G4 ratio	0.04	−0.47	−0.75	0.17	0.64	0.56
β-glucan peak area	0.51	−0.14	0.47	−0.07	−0.34	−0.29
						
*Wholemeal fibre components*						
Tot-AX	0.16	0.02	0.15	1.00	0.26	–
WE-AX	0.57	−0.24	0.01	0.26	1.00	–
AX solubility	0.53	−0.25	−0.04	−0.06	0.94	–
%x	−0.06	0.22	0.24	−0.12	−0.16	–
%x_2_	0.08	−0.44	−0.47	0.16	0.22	–
%x_3_	−0.08	−0.07	0.07	−0.06	0.03	–
%x_5_	0.18	0.36	0.41	0.30	0.05	–
%xa^3^xx	−0.21	0.39	0.49	−0.15	−0.23	–
%xa^2+3^xx	−0.01	0.52	0.30	−0.28	−0.31	–
%xa^3^a^3^xx	−0.42	0.39	0.44	0.04	−0.31	–
%xa^3^xa^3^xx	0.01	−0.04	−0.13	−0.29	−0.12	–
%xa^3^a^2+3^xx	0.18	0.35	0.35	−0.19	−0.13	–
%xa^3^xa^2+3^xx	0.13	0.27	0.31	−0.25	−0.05	–
G3: G4 ratio	0.18	−0.38	−0.83	−0.13	0.10	–
β-glucan peak area	0.33	0.13	0.55	−0.07	0.11	–

YR, year of release; TKE, thousand kernel weight; TW, test weight; Tot-AX, total arabinoxylan; WE-AX, water-extractable arabinoxylan; RV, relative viscosity.

## 2 Experimental design, materials and methods

### 2.1 Plant material

Grain samples from fifteen Italian durum wheat *(Triticum turgidum spp. durum)* genotypes, comprising four old landraces (Dauno III, old Saragolla, Russello, Timilia RB), three old cultivars (Cappelli, Garigliano and Grifoni 235) and eight modern cultivars bred after 1985 were analysed. These were obtained from the same two field trial (in 2013 and 2014) as reported in [2], but separate samples of grain were analysed. Plants were grown in a randomized complete block design with three replications on a clay–loam soil at Foggia (Italy, 41° 28′ N, 15° 32′ E and 75 m a.s.l.), as reported previously [2]. The two crop seasons were characterized by different amounts of rainfall during the grain development stage (54 mm and 153 mm respectively in 2013 and 2014).

Wholemeal and semolina flours were prepared using a Cyclotec Tecator 1093 sample mill (sieve 1 mm) and a laboratory mill (Bona, 4 cylinders, sieve 180 µm), respectively. Ash was determined by NIR using an Infratech 1241 Analyser (Foss, Hillerod, Denmark). Nitrogen was determined using the Dumas combustion method using a CNS Combustion Analyser (Leco Corp., St Paul, MN, USA) and % protein calculated as % N×5.7.

### 2.2 Enzyme fingerprinting of arabinoxylan and β-glucan

Enzyme fingerprinting of AX and β-glucan was as described by [3]. 100 mg aliquots of semolina and wholemeal flours were digested with endo 1,4 β-xylanase (E.C.3.2.1.8) (a xylanase of the GH11 group) and endo 1,3(4) glucanase (lichenase) (E.C.3.2.1.73) (both enzymes from Megazyme, Bray, Ireland) to digest arabinoxylan and β-glucan, respectively. The oligosaccharides were separated by HP-AEC and the peak areas of the arabinoxylan oligosaccharides (AXOS) were expressed as percentages of the total peak areas of all AXOS. The two major gluco-oligosaccharides (GOS) released by enzymatic digestion of β-glucan by lichenase comprised three glucose residues (G3) and four glucose residues (G4). Total β-glucan was therefore calculated as the sum of the G3 + G4 peak areas and the ratio of G3 to G4 fragments calculated.

### 2.3 Relative viscosity

Aqueous extracts were prepared from semolina as described by [4] but with an additional centrifugation step at 10,000 × *g* for 10 min at room temperature before filtration. They were stored on ice prior to measurement of relative viscosity (ηrel = t/t0, where t0: flow time of distilled water, 72–74 s) at 30 °C using an automated viscometer (AVS 370, SI Analytics, Germany) fitted with an Ostwald capillary tube (2 ml, diameter 0.4 mm). Values are the means of two extractions with the flow time of each extract being measured five times.

### 2.4 Statistical analysis

Two-way analysis of variance (ANOVA) was carried out using as factors genotype and crop season. Least significant difference (LSD) was used at P≤0.05. ANOVA and correlation analyses were performed with software JMP (Version 8.0.2, SAS Institute Inc., 2009).
