# Supplementary material for: Data set of enzyme fingerprinting of dietary fibre components (arabinoxylan and β-glucan) in old and modern Italian durum wheat genotypes
Source: Data Brief. 2017 Dec 19;16:1062–8. doi: 10.1016/j.dib.2017.12.029 (PMC5760467; doi:10.1016/j.dib.2017.12.029)
Supplement: Supplementary file 1 — Supplementary material [file mmc1.docx]

Conflict of interest: Data set of enzyme fingerprinting of dietary fibre components (arabinoxylan and β-glucan) in old and modern Italian durum wheat genotypes

The authors declare no conflicting interests

**
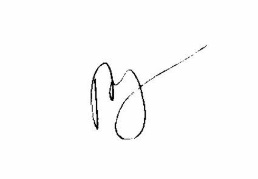
**

Peter Shewry, 5 Dec 2017
